# 

**Published:** 1841-12

**Authors:** 


### WESTERN JOURNAL

OF

### MEDICINE AND SURGERY.

### COLLABORATORS.

Edward H. Barton, M. D. *late Professor of the Theory and Practice of Medicine, in the Medical College of Louisiana.*

William J. Barbee, M. D. *Cincinnati, Ohio.*

George W. Bayless, M. D. *Demonstrator of Anatomy in the Louisville Medical Institute.*

Solon Borland, M. D., *Memphis, Tennessee.*

A. H. Buchanan, M D. *Columbia, Tennessee.*

Charles Caldwell, M. D. *Professor of the Institutes of Medicine and Medical Jurisprudence in the Louisville Medical Institute.*

Samuel A. Cartwright, M D. *Mississippi.*

A. Clapp, M. D. *New Albany, Indiana.*

Jedediah Cobb, M. D. *Professor of Anatomy in the Louisville Medical Institute.*

Thomas W. Colescott, M. D. *Louisville, Ky.*

John Esten Cooke, M. D. *Professor of the Theory and Practice of Medicine in the Louisville Medical Institute.*

Samuel D. Gross, M. D. *Professor of Surgery in the Louisville Medical Institute.*

John Hardin, M. D. *Munfordsville, Ky.*

John P. Harrison, M. D. *Professor of Materia Medica, in the Medical College of Ohio.*

John F. Henry, M. D. *Bloomington Illinois*

Samuel P. Hildreth, M. D. *Marietta, Ohio.*

Samuel Hogg, M. D. *Nashville, Tennessee.*

M. Z. Kreider, M. D., *Lancaster, Ohio.*

Moses L. Linton, M. D. *Springfield, Kentucky.*

Henry Miller, M. D. *Professor of Obstetrics and the Diseases of Women and Children in the Louisville Medical Institute.*

John W. Monette, M. D. *Washington, Mississsippi.*

Samuel B. Richardson, M. D., *Lecturer on Anatomy and Surgery, &c, Louisville, Ky.*

John L. Riddell, M. D. *Professor of Chemistry in the Medical College of Louisiana.*

Landon C. Rives, M. D. *late Professor of Obstetrics in the Medical Department of the Cincinnati College.*

Charles W. Short, M. D. *Professor of Materia Medica in the Louisville Medical Institute.*

G. Troost, M. D. *Professor of Chemistry and Mineralogy in the University of Nashville*

Amasa L. Trowbridge, M. D. *Professor of Surgery, Willoughby University, Ohio.*

John A. Warder, M. D. *Cincinnati, Ohio.*

William Wood, M. D. *Cincinnati, Ohio*

### TO CORRESPONDENTS.

Dr. Ferguson's Pathological and Clinical Reports, Dr. Carroll's paper on the Topography and Diseases of the Eastern part of Ohio, Dr. Smick's paper on those of the Wabash valley, Dr. Jones on Digitalis, and Dr. Dawson on Scarlatina, with several other communications have been unavoidably postponed.

### INDEX TO VOL. IV.

A

Abdominal disease, fatal case of, 34
Abdominal disease, case of, - 260
Albumen, value of in urine, - 302
Anus, fissure of, - - - - - 215
Ano, fistula in, - - - - - 318
Aorta, compression of, - - 209
Arsenic, sold for Antimony, - 405
Arteries of the arm, wound of, - 33
Ascending aorta, wound of, - 146
Axillary aneurism, case of, - - 205
Artery carotid, ligature of, - - 148
Asylums, Lunatic, - - - - 443

B

Bilious fever, prize essay on, - 161
Bloody murrain, - - - - 268
Bubo, treatment of, - - - - 241
Bibliographical notices, - - 51
" " - - - - 52
" - - - - - 294
Board of Health, of New Orleans, 315

C

Calculus, effects of, - - - - 396
Calculous concretion, case of, - 300
Carotid Artery, ligature of, - 148
Cattle, murrain of, - - - - 407
Cavity of thorax, effusions into, - 72
Cholera infantum, ice in, - - 366
Climate of the United States, - 487
Congestive fever, sulph. quin., - 402
" " Sinapisms in, 487
Cooper, Sir A. P., last illness of, - 65
Coup de soliel, - - - - - 317
Creosote, employment of, - - 394
Conium and iodine, inhalations of, in phthisis pulmonalis, - - 375

D

Disunited fracture, - - - - 97
Dysentery, endemic, - - - 315
Diaphragmatic hernia, - - - 14
Dropsy, ovarian, - - - - 309
Dislocation and fracture cases of, 106

E

Electro-magnetism, in neuralgia, - 378
Endemic dysentery, - - - 315
Ergot, observations on, - - 53
" " - - - - - 374
" " - - - - - 380

F

Fallopian tube, rupture of, - - 141
Fever, - - - - - - 77
Fever, intermittent, tubercles developed by, - - - - - 150
Fistula, in ano, - - - - 318
Fracture and dislocation, cases of, 106
Fever, yellow, of Natchez, - - 311
" " " - - 411
Fungus Hæmatodes, - - - 440

G

Genio-hyo-glossi muscles, division of, - - - - - - - 395
Gun shot wound, case of, - - 28

H

Hairs discharged from the thumb, 483
Hernia, diaphragmatic, - - 44
Heart, wound of, - - - 144
Hospitals, lunatic, - - - 443
Hunter, John, sketch of, - - 37
Hydrocele, new cure for, - - 76
Hydrocele, objections to operations for, - - - - - - - 100
Hydrocele, case of, - - - 107
Hydrocele, new treatment of, - 313

I

Imperforate vagina, operation for, 110
Improvement medical profession, address on, - - - - - 81
Institution, orthopœdic, - - 233
Inferior vena cava, obliteration of, 142
Intermittent fever, tubercles developed by, - - - - - - 159
Irritable eye, belladonna in - 373
Irritation, death by, - - - 398
Iodine and conium, inhalations of, 375

J

Journal medical, - - - - 320
Journal, consultations through, - 404
Journal, publication of medical books with, - - - - - 399

K

Kentucky, State Medical Society of, 316
Kentucky Lunatic Asylum, - 467

L

Lacteal secretion, absence of, - 317
Liver, resistance of to putrefaction, 233
Leviathan, - - - - - - 149
Lithotomy, - - - - - - 159
Louisville, Medical Institute of, - 160
" " " - - 238
" " " - - 410
" " " - - 487

M

Magendie's method of treating neuralgia, - - - - - - 378
Maladies ventreses, traite de, - 111
Milk-sickness, treatment of, - 229
Medical profession improvement in, 81
Milk-sickness, morbid anatomy of, 234

Morbid anatomy of milk-sickness, 234
Milk-sickness, treatise on, - 308
Medical schools, - - - - 228
Museum pathological, contributions to, - - - - - - 73
Medicine in Paris, - - - 237
Medical Institute of Louisville, - 160
" " " - - 228
" " " - - 410
" " " - - 487
Murrain of cattle, - - - 268
" " - - - - - 408
Muscles, genio-hyo-glossi, division of, - - - - - - - 385

N

New Orleans, board of health of, - 315
Neuralgic affections, stramonium in, 229
Nitrate of potassa, vapors of, - 158
Notices, bibliographical, - - 51
" " - - - - 291
Neuralgic diseases, electricity in, 409
Neuralgia, Magendie's mode of treating, - - - - - 378
National institution, proceedings of, 372

O

Obituary, - - - - - - 80
Ovarian dropsy, case of, - - 309

P

Paris, medicine in, - - - 237
Pathological museum, contributions to, - - - - - - - 73
Plethora, remarks on, - - - 1
Poisoning with whiskey, symptoms of, - - - - - - - 230
Physiology and animal mechanism, 371
Potassium, iodide of, - - - 398
Polypus uteri, case of, - - 295
Prize essay on fever, - - 227
Professor Wagner, death of, - 157
Proceedings of National Institution, 372
Phthisis pulmonalis, iodine, new method, - - - - - 364
Pulmonary and rheumatic diseases, statistics of, - - - - 271
Progress of science, - - - 400

R

Rheumatism, treatment of - - 224
Re-vaccination in deaf and dumb institution, result of, - - - 138

S

Satterwhite, death of, - - - 409
Science, progress of, - - - 400
Scientific hoax, - - - - 320
Scrofula, cases of, - - - 361
Shower of flesh and blood, - - 485
South Carolina, medical school, - 156
Solar asphyxia, - - - - 384
Spinal irritation, case of, - - 363
Spinal column, - - - - 356
Stammering, - - - - 236
Stammering, operation for, - 125
Stricture of urethra, - - - 305

T

Tenotomy, - - - - 80
Tennessee medical society, - - 157
Thorax, effusions into, - - 72
Tinea capitis, formulæ used in, 375
Thumb, dislocation of - - 313
Tennessee Lunatic Asylum, - 408
Tetanus, cured by section of nerve 314
Tonsil, excision of, - . - 299
Transylvauia University, - - 80
Tubercular phthisis, inhalations in, 375

U

Uterus, extraction of foreign body from, - - - - - - 225
Uvula end tonsil, excision of, . 229
Urticaria, - - - - - 403
Urine, value of albumen in, - 302
University, Transylvania, - - 80

V

Vaccination and small pox, observations on, - - - - - 135
Vaccination and re-vaccination, general results of, - - - 139
Vena cava, obliteration of, - - 142
Vagina imperforate, operation for 110

W

Weather, - - - - - 160

Y

Yellow fever, observations on, 331
" " " - - 411

### RECEIPTS

*For the Medical Journal for December*, 1841.

---

| | | |
|---|---|---|
| Dr. | J. X. O. Haver, Carlisle, Ia., - - - - | $5 00 |
| | W. Fennell, Cottonville, Ala., - - - - | 5 00 |
| | H. G. Duerson, Westport, Ky., - - - - | 5 00 |
| | G. R. Wharton, Huntsville, Ala., - - - | 5 00 |
| | Kirkpatrick, Rural Hill, Tenn., - - - - | 5 00 |
| | T. Goodall, Tompkinsville, Ky., - - - | 7 75 |
| | G. B. Walker, Evansville, Ia., - . - - | 10 00 |
| | J. C. Taylor, County - - - - - - | 5 00 |
| | S. Fleming, Franklin, Tenn., - - - - | 7 00 |
| | A. Quarles, Frankfort, Ky., - - - - | 10 00 |
| | J. R. Desha, Little Rock, Ark., - - - - | 5 00 |
| | P. M. Keith, Merom, Ia., - - - - - | 5 00 |
| | N. F. Scales, Lewisburg, Tenn., - - - - | 5 00 |
| | J. Slemons, Corydon, Ia., - - - - - | 5 00 |
| | E. W. Harris, Jackson, Mo. - - - - | 5 00 |
| | G. Holcomb, Hague, Ky. - - - - - | 5 00 |
| | W. J. Goulding, Little Rock, Ark., - - - | 5 00 |
| | G. G. Boyle, Providence, Ky., - - - - | 5 00 |
| | M. Johnson, Roscoe, O., - - - - - | 5 00 |
| | J. Harris, Salem, O., - - . - - - | 5 00 |
| | T. A. Woodward, City, - - - - - | 5 00 |
| | R. G. P. White, Pulaski, Tenn., - - - - | 6 00 |
| Prof. | A. Moore, Claysville, Ala., - - - - | 10 00 |
| Dr. | J. H. Stewart, Bellefontaine, Ala. - - - | 3 00 |
| | J. M. Riley, Orleans, Ia. - - - - - | 5 00 |
| | W. R. Dulaney, Blountsville, Tenn., - - | 5 00 |
| | Fred. Jones, Wheeling, Miss., - - - - | 1 00 |
| | J. M. Whitsell, Knightstown, Ia., - - - | 8 00 |
| | G. W. C. Bond, Huddleston's Cross Roads, Tenn., | 5 00 |
| | A. Leslie, Petersburg, Ia., - - - - - | 5 00 |

### TO SUBSCRIBERS.

Another volume of this Journal is concluded by this number. We find, on looking over our books, that the arrearages are very numerous, and we call upon subscribers to remit by the commencement of the new year. We find the number of those who have not paid for the first year so small, that we shall endeavor to give a complete list of their names in the February number. We hope that subscribers generally, those who owe no arrearages as well as those who do, will remit about the first of the coming year. Before we send out the January number of the new year, we must purge our list.

PRENTICE & WEISSINGER.

The WESTERN JOURNAL OF MEDICINE and SURGERY is published monthly by the undersigned, at the corner of Main and Fifth streets, Louisville, at $5 per annum, payable in advance. Each number contains from 80 to 84 pages making two volumes in the year of about 500 pages.

Contributions to its pages by the Physicians of the Valley of the Mississippi, addressed to the Editors, are respectfully solicited.

Letters on business, to be addressed, postage paid, to the publishers.

☞Postmasters, by the regulations of the Postoffice Department, will frank letters containing subscription money, and all remittances so franked are at the risk of the publishers.

*July* 25, 1841. PRENTICE & WEISSINGER.

